# Development of a Sham Smartphone App for Opioid Use Disorder: Acceptability and Suitability Study

**DOI:** 10.2196/71105

**Published:** 2025-08-08

**Authors:** Kierstyn S Gallegos, Jennifer S Potter, Van L King, Gregg Siegel, Leslie H Siegel, Elise N Marino

**Affiliations:** 1 Be Well Institute on Substance Use and Related Disorders, University of Texas Health Science Center at San Antonio, 5109 Medical Drive, San Antonio, TX, 78229, United States, 1 210 450 3760; 2 Department of Psychiatry and Behavioral Sciences, University of Texas Health Science Center at San Antonio, San Antonio, TX, United States; 3 Biomedical Development Corporation, San Antonio, TX, United States

**Keywords:** prescription digital therapeutic, sham software, believable, placebo, digital health

## Abstract

**Background:**

Despite having evidence-based medication for opioid use disorder (OUD), dropout is one of the most common issues noted with this treatment. Prescription digital therapeutics, which are app-based interventions prescribed by a health care professional, have the potential to increase adherence to medication for OUD and retention while overcoming treatment barriers, including provider capacity and patient access. Using a sham app as a control condition for a randomized clinical trial is an innovative method to establish the true efficacy of these apps.

**Objective:**

This study included the development and testing of a sham smartphone app for OUD.

**Methods:**

After the sham app was developed, participants were enrolled in a 4-week trial examining the use and suitability of the sham app as a control condition. Criteria for determining suitability included (1) participants believing the sham app is an active intervention and (2) participants experiencing no clinical improvements in depression severity or quality of life after using the sham app. Self-reported depression severity and quality of life were captured before and after using the sham app. A user satisfaction survey and semistructured interviews were conducted at the end of the study. Quantitative analyses included paired 2-tailed *t* tests. The semistructured interviews were conducted with 20 of the 21 participants, and these interviews were analyzed using rapid qualitative analysis.

**Results:**

Overall, 21 participants (mean_age_ 42.0, SD 6.4 years; female: n=9, 43% and male: n=12, 57%) were enrolled. The average number of app log-ins was 17.8 (SD 10.6; range 1-41). There were 2 participants who only logged in 1 time, and 15 (71%) participants completed the goal of logging in an average of 3 times per week. No significant differences were found in depression severity (*P*=.50) or quality of life (quality of life: *P*=.42, physical health: *P*=.58, psychological health: *P=.*07, environmental health: *P*=.44, and social relationships: *P*=.86) after using the sham app. Of the 20 participants who completed the semistructured interview, 19 (95%) believed that they were using an active intervention. The user satisfaction survey revealed high overall satisfaction with the sham app with a score of 91%. Qualitative analyses revealed several recurring themes, including perceived value and impact, potential for behavior change, use patterns and engagement, perspective and usability, and perceptions of authenticity.

**Conclusions:**

Our sham app met our a priori criteria for suitability as a sham app. No *c*linical improvements from baseline were observed at the end of the study period, and all but 1 participant believed that they were using an active intervention. Demonstrating that this sham app is suitable as a control condition elevates the rigor of randomized clinical trials and ensures the efficacy of prescription digital therapeutics.

## Introduction

In 2017, the estimated total economic burden of opioid use disorder (OUD) and opioid-related overdose deaths in the United States was US $1.02 trillion [1,2]. The high prevalence of OUD [3] coupled with the significant health care cost of treating it has resulted in the need for an innovative and cost-effective approach to care that will complement existing evidence-based treatment.

Medication for opioid use disorder (MOUD), including methadone, buprenorphine, and naltrexone, is standard of care with the latter 2 available in office-based settings [4]. Despite robust evidence demonstrating the effectiveness of MOUD [5], there are limitations to receiving this in-office treatment [6]. Wait times are often extended due to the availability of providers working within standard clinic operating hours [7]. Such delays in treatment are detrimental and potentially fatal [8]. Being unable to receive care in times of crisis and a strictly regulated time allotted for patient sessions [9] are significant barriers to entering and maintaining engagement in treatment, creating potential gaps in service [10]. Reliable transportation, childcare, and clinic proximity also serve as barriers.

Overcoming these barriers to receiving in-office treatment necessitates enhancing MOUD treatment with evidence-based interventions that are not limited by clinician availability or patient access. App-based interventions, which are easily accessible and available at any time, have demonstrated efficacy for those with OUD [11-14]. Prescription digital therapeutics are app-based interventions that are used to treat or manage physical or mental health conditions, such as OUD. They can be used as monotherapy or as an adjunctive treatment. Unique from other app-based interventions, prescription digital therapeutics must be prescribed by a health care professional and are eligible for reimbursement from some health insurance companies. To receive this designation, a prescription digital therapeutic must receive approval from the US Food and Drug Administration (FDA) after demonstrating its safety and efficacy in a clinical trial. These apps are empirically validated interventions, a classification not attributed to most freely available apps, which increases the likelihood that patients will benefit from them. Although prescription digital therapeutics require a prescription and thus at least 1 clinician visit, once given access to the app, patients are not limited in their engagement with the intervention.

In 2018, the FDA approved reSET-O, an 84-day prescription digital therapeutic, to improve retention in outpatient treatment for OUD [15]. reSET-O received FDA approval after the completion of a randomized trial that used treatment as usual for the control condition. In doing so, the efficacy demonstrated by reSET-O may be attributable to having access to an app rather than the intervention provided within reSET-O itself. This uncertainty is inherent in an unblinded study design, highlighting the essential role of using a sham app.

A sham condition should be indistinguishable from the experimental condition in appearance and functionality. It must also provide no therapeutic benefit to serve as an acceptable control [16]. Distinct from drug trials that use an inactive substance, simply engaging with a digital platform poses risk for measurable improvement [17], making the development of a sham app much more challenging. The magnitude of naming an app-based intervention an FDA-approved prescription digital therapeutic nevertheless requires a blinded study design.

Ensuring the efficacy of prescription digital therapeutics is paramount to the complexities of designing a sham app. Recognizing this, the field is evolving. One of the first to use a sham condition in pursuit of receiving FDA approval as a prescription digital therapeutic is KIOS, an innovative app that was developed using contextual design methodology to augment MOUD treatment [16]. As opposed to the module-based intervention provided by reSET-O, the KIOS app delivers customized, patient-driven behavioral interventions in real time [9].

The study described here was designed to test the suitability of the KIOS sham app as a control condition for a randomized trial. A priori criteria for determining suitability included (1) participants believing the sham app is an active intervention and (2) participants experiencing no clinical improvements in depression severity or quality of life after using the sham app. Being among the first to develop a sham app, we are transforming the standard of clinical trials and consequently prescription digital therapeutics.

## Methods

### Overview

This study used a mixed methods design to evaluate whether our sham app could serve as a believable control condition in a randomized controlled trial as well as to evaluate participant perspectives of usability and satisfaction with the app.

### Ethical Considerations

This fully web-based study was approved by the University of Texas Health Science Center at San Antonio Institutional Review Board (protocol 20230232HU). Participants provided written informed consent prior to participation. During the consent process that was conducted remotely, participants were informed that the purpose of the study was to evaluate engagement, usability, and user satisfaction with a mobile app that offered information about OUD and other substance use disorders. Participants received US $200 for their participation in the form of a ClinCard MasterCard. Compensation was automatically credited after completion of the study. All data were deidentified prior to being analyzed.

### Participants

Participants were all diagnosed with OUD, currently receiving MOUD, and in stable outpatient treatment for at least 4 weeks at the time of enrollment. They were recruited from outpatient MOUD treatment facilities in Texas. Recruitment strategies included placing flyers in clinic waiting rooms, announcements during web-based trainings sponsored by our staff, sending emails about the study to our network of MOUD providers, and outreach personnel speaking with people in line at methadone clinics. Individuals interested in participating in the study were directed to contact study staff via phone. Inclusion criteria for the study included (1) male or female outpatients 18 years of age or older; (2) diagnosis of OUD; (3) currently stable in OUD outpatient treatment, defined as medication adherence and attending outpatient appointments for 4 weeks or longer; and (4) ability to access the KIOS sham app via a smartphone or tablet. Participants were excluded if they (1) were unwilling or unable to comply with study requirements, (2) had a psychiatric or medical disorder interfering with the ability to use the app, (3) were incarcerated, or (4) were pregnant.

### Measures

The Patient Health Questionnaire-9 (PHQ-9) is a 9-item validated self-report assessment that was used to measure depression severity over the past 2 weeks [18]. The World Health Organization Quality of Life-BREF (WHOQOL-BREF) assessment was used to assess self-reported quality of life across 4 domains: physical health, psychological health, social relationships, and environment over the past 2 weeks [19]. A User Satisfaction Survey was administered to assess the degree of satisfaction with the sham app [20]. The WHOQOL-BREF and PHQ-9 were chosen as outcomes because they assess complementary facets of recovery (eg, depression has a high comorbidity with alcohol and drug use) [21-23]. Improvements in depression and quality of life are some potential benefits that may arise when using an app-based intervention; thus, treatment targets for the app are not limited to opioid use but rather to all aspects of recovery that may impact opioid use and retention in MOUD treatment.

### Procedure

As this was a web-based study, communication between research assistants and participants occurred via telephone and Zoom (Zoom Video Communications). No changes were made to the app after the trial began.

This was a 1-sample study with no control group using a pre- and posttest design. Prior to engaging with the sham app, participants completed the PHQ-9 and WHOQOL-BREF delivered via an electronic data capture system. Surveys were completed while on the phone with research assistants in the event that participants had any questions. They were then given access to the app for 4 weeks, during which time they were instructed to engage with the app at a minimum of 3 times per week and as often as once daily. Participants were encouraged to explore all the functions within the app. At the end of the 4 weeks, they completed the PHQ-9 and WHOQOL-BREF as well as the User Satisfaction Survey.

Upon completion of the study, semistructured interviews were conducted to gather detailed information on believability, usability, and satisfaction with the sham app. Six questions were asked assessing app believability and satisfaction (eg, “Tell us what you thought of KIOS?” and “If you weren’t in the study anymore, would you use the app? Why or why not?”). Following these questions, a debriefing statement was delivered informing participants of the sham nature of the app. Once informed, participants were asked to reshare their perception of the sham app and indicate whether they suspected it was anything other than what it was presented as.

### Sham App Development

The sham app was developed for use in a randomized controlled trial testing the KIOS app, and it was created at the request of the FDA. The proposed design of the sham app was presented to the FDA for review prior to proceeding with development.

The sham app was meticulously designed to convince the user that it was an active intervention despite being designed to have no therapeutic value. To create a believable sham app, the active KIOS design was replicated to create a visually similar version that lacked key therapeutic components. The nonspecific factor of the sham app mirrored the active condition by replicating the layout and functionality of account log-in, color palette, button shapes, emergency contact functions, and landing pages. Features that incorporated elements of an active intervention were removed from the sham and were replaced with general drug and alcohol educational content taken from the National Institute on Drug Abuse (NIDA).

We identified, selected, and analyzed information about drugs using specific criteria. Drug-related facts were explored through key questions addressing the drug’s definition, use, short- and long-term effects, physical and chemical properties, and health impacts. Questions using a true or false format were included in the app, and they were designed to generate generic feedback that was presented to participants. These questions were developed by selecting topics from the NIDA website, identifying relevant passages, and rephrasing or editing them for clarity and conciseness. Correct “true” answers were based on accurate passages, while “false” answers included explanations derived from the content available from NIDA. The process also involved assessing the grade level of each question to ensure readability at or below a sixth-grade reading level, excluding drug names, and documenting both the grade level and source for each question. Participants were presented with the true or false questions 3 times a week followed by questions assessing their level of interest in the educational content being provided. Because education alone is unlikely to change long-term behavior [24], the final product was a fully functional, education-based learning tool that had no active components capable of creating meaningful improvements in clinical outcomes.

The second fundamental difference between the active and sham versions of KIOS is the reminder push notification. The push notification frequency was customizable to the user’s preference, ranging from daily, every other day, to not at all. The active KIOS app that was developed is a tailored, patient-centered app, thus necessitating a similar approach to the reminder notifications. Participants using the active version receive personalized reminders (eg, “Savor this one moment with a slow, deep breath. Then, connect with yourself further in KIOS”). In contrast, the sham app was designed to be standardized across users, including the reminders that used generic language (eg, “This is the KIOS reminder you set for yourself”). This difference is essential because the personalized reminders are designed to simulate the therapeutic intent of an active intervention while generic reminders do not.

### Statistical Analysis

Quantitative analyses included paired 2-tailed *t* tests to assess differences in PHQ-9 and WHOQOL-BREF scores after using the sham app. To determine overall satisfaction with the app, a total percentage of the aggregate responses of the User Satisfaction Survey was calculated.

Rapid qualitative analysis was used to systematically review the semistructured interviews that were conducted following completion of the study. Transcripts were thoroughly reviewed to identify recurring themes. Each question was separated with participant responses to the question being condensed into bullet points. After grouping the questions into overarching domains (eg, use patterns and engagement and perceptions of authenticity), recurrent themes were cataloged within the domains to capture larger research focus areas. The final summary template that was used to aggregate participant responses was standardized across participants to ensure consistency.

A matrix was then developed to display the condensed summaries. The columns were organized by domain with subsections for each question in the domain. Each row in the matrix was dedicated to a participant interview. The matrix was filled using the data from the summaries and analyzed across rows and down columns to identify central themes, allowing for a comprehensive understanding of key findings from the interviews. The qualitative analysis was completed manually, and the quantitative analyses were completed in Microsoft Excel.

## Results

Consistent with our recruitment goal, 21 participants provided informed consent, used the app during the 4-week trial, and completed all pre- and postassessments. The average age was 42.0 (SD 6.4) years, 9 (43%) were female, and 12 (57%) were male.

**Figure 1.**
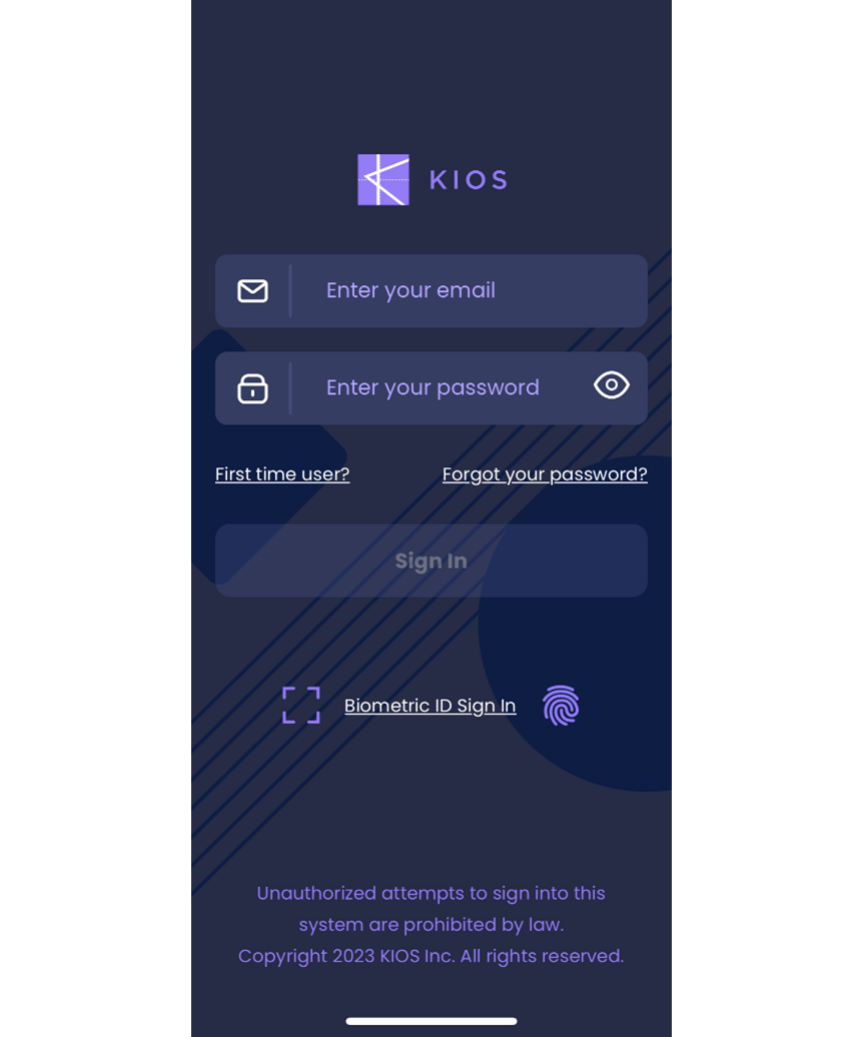
App log-in landing page.

Among the full sample, the average number of app log-ins was 17.8 (SD 10.6; range 1-41). There were 2 participants who only logged in 1 time, and the rest of the sample logged in a minimum of 6 times. A total of 15 (71%) participants completed the goal of logging in an average of 3 times per week. Figure 1 displays the app log-in landing page. Quantitative analyses indicated that there were no statistically significant differences in depression severity or quality of life from pre- to postapp use (Table 1). The User Satisfaction Survey indicated high overall satisfaction with the sham app, with an average score of 91%.

Of the full sample, 20 (95%) completed the end of study semistructured interview. Qualitative analysis of these 20 participants revealed several recurring themes, including perceived value and impact, potential for behavior change, use patterns and engagement, perspective and usability, and perceptions of authenticity (Table 2). Participants characterized the sham app as simple, user-friendly, and educational. Participants also reported high satisfaction with the software and increased app use, as they became more familiar with it. Participants valued the educational content and increased awareness of drug-related health risks. There was a notable desire among users to retain access to the software beyond the study. Despite positive feedback, most participants did not believe that the sham app would change their substance use.

In total, 19 of 20 (95%) participants reported during the semistructured interview that they believed they were enrolled in an active intervention.

**Table 1.** Bivariate analyses examining depression severity and quality of life from pre- to postuse of the sham app.

Measure	Mean (SD)	Coefficient	*P* value
PHQ-9^a^		2.1	.50
Pre	10.7 (37.6)		
Post	11.8 (33.2)		
WHOQOL-BREF^b^			
Quality of Life		2.1	.42
Pre	66.7 (411.5)		
Post	69.6 (415.2)		
Physical health		2.1	.58
Pre	56.6 (255.5)		
Post	55.1 (263.5)		
Psychological health		2.1	.07
Pre	64.9 (209.3)		
Post	58.9 (446.7)		
Environmental health		2.1	.44
Pre	59.7 (338.8)		
Post	56.7 (481.7)		
Social relationships		2.1	.86
Pre	57.1 (606.2)		
Post	56.3 (693.8)		

a PHQ-9: Patient Health Questionnaire-9.

b WHOQOL-BREF: World Health Organization Quality of Life-BREF.

**Table 2.** Qualitative themes and quotes from participants about their perception of the sham app following software engagement.

Theme	Representative quotations
Perceived value and impact	“I took it as a learning process… like the long-term effects like people catching the hepatitis, HIV, blood clots, high blood pressure. And that stuck in my head.” “Like learning, learning certain things that I’ve never thought I would.”
Potential for behavioral change	“The version that I used to if I was a full drug blown back into drugs again would that help me in trying to recover? No.” “I don’t think it changed the way I was or the way I am.”
Use patterns and engagement	“I found myself wanting to know more ... I found myself using it more and more often.” “I would say I would still use it because of the information.”
Perspective and usability	“... it was super user friendly and some of the questions that were I was I was not expecting.” “It was like no difficulty with it at all. Like literally.”
Perceptions of authenticity	“I didn’t know that there was more to it, but I mean, I’m glad.” “I thought that was it, you know, I mean. I didn’t think it was gonna be anything different.”

## Discussion

### Principal Findings

Our findings indicated no significant change in self-reported depression severity or quality of life after using the sham app, and all but 1 participant believed that the app was an active intervention. Together, these findings meet our a priori criteria that the sham app appears to be a suitable control condition for a randomized trial.

Prescription digital therapeutics should be held to the highest standard of research. This makes blinded research designs crucial for determining true efficacy before prescribing these apps to patients. Because a randomized controlled trial is the optimal method for testing the evidence base of a treatment, a sham app should be expected. The creation of a sham app for testing a prescription digital therapeutic is a relatively new and modernizing advancement in research designs [25]. Recognized by the FDA as a uniquely difficult undertaking [17], this novel approach addresses the need for a more scientifically robust methodology in the study of prescription digital therapeutics.

With an appealing user interface, the design and execution of the sham software were pivotal in presenting the app to be nearly indistinguishable from an active intervention. The sham app was reported to be engaging, yet nontherapeutic. The latter signifies the possibility to surmount the potential benefits inherent in engaging with an interactive digital platform. The identical user interface, testing prior to using it in a randomized controlled trial, and positive engagement results we demonstrated here overcome previously cited limitations in the study and use of sham apps [25]. This sham app holds significant potential for OUD treatment and recovery, paving the way for creating studies to identify effective prescription digital therapeutics. Without it, the gap between OUD and a good prognosis will remain.

The success of the sham app extends beyond the immediate study, and it represents an opportunity to elevate the standards of study designs for all digital health research. Using the framework we have demonstrated here can further the development of future sham apps. We have shown some components of a sham app that make it believable without producing clinical improvements (eg, generic push reminders and using education in place of the active intervention). These components can be modified and adapted for future studies testing app interventions, making our framework appropriate for creating a sham app that will be suitable as a control condition. This innovation holds significant implications not only for KIOS but also for the broader research field, advancing the science, testing, and deployment of prescription digital therapeutics.

### Limitations

There are limitations that should be mentioned. This study included a small sample size, and the study duration was short. Because our initial pilot study demonstrated the feasibility and acceptability of the KIOS app and this study was designed to create a comparison, sham app for future testing of the KIOS app, we used a pre- and posttest design with no control group [9]. Participants were not required to see a medical provider to be included, and we do not have information regarding their opioid use or adherence to their medication regimen. There may have been variations in interview techniques among researchers. It is possible that a sham app may have some therapeutic benefits among those early in recovery. Finally, all participants were in Texas, making additional research in other geographic regions necessary. Despite these caveats, the insight gained here is invaluable.

### Conclusions

With the rapid integration of technology in health care, there has been a call for more methodologically rigorous studies. Developing this sham app marks a historic accomplishment in the emerging field of prescription digital therapeutics. This advancement will drive high-quality research, providing the foundation for a new placebo that will revolutionize the future of clinical trials.

## Abbreviations

FDA: US Food and Drug Administration
MOUD: medication for opioid use disorder
NIDA: National Institute on Drug Abuse
OUD: opioid use disorder
PHQ-9: Patient Health Questionnaire-9
WHOQOL-BREF: World Health Organization Quality of Life-BREF
